# Green biosynthesis of bimetallic ZnO@AuNPs with its formulation into cellulose derivative: biological and environmental applications

**DOI:** 10.1186/s40643-024-00759-3

**Published:** 2024-06-17

**Authors:** Mohamed A. Al Abboud, Abdullah Mashraqi, Husam Qanash, Hattan S. Gattan, Hashim R. Felemban, Faeza Alkorbi, Mohamed M. Alawlaqi, Tarek M. Abdelghany, Hanan Moawad

**Affiliations:** 1 Biology Department, College of Science, Jazan University, 82817 Jazan, Saudi Arabia; 2 Department of Medical Laboratory Science, College of Applied Medical Sciences, University of Ha’il, 55476 Hail, Saudi Arabia; 3 Medical and Diagnostic Research Center, University of Ha’il, 55473 Hail, Saudi Arabia; 4 Department of Medical Laboratory Sciences, Faculty of Applied Medical Sciences, King Abdulaziz University, 22254 Jeddah, Saudi Arabia; 5 Special Infectious Agents Unit-BSL3, King Fahd Medical Research Center, King Abdulaziz University, 21362 Jeddah, Saudi Arabia; 6 Department of chemistry, Faculty of Science and Arts at Sharurah, Najran University, 68342 Sharurah, Saudi Arabia; 7 Botany and Microbiology Department, Faculty of Science, Al-Azhar University, Cairo, 11725 Egypt; 8 Plant Department, Faculty of Science, Fayoum University, Fayoum, 63514 Egypt

**Keywords:** Bimetallic nanoparticle, Cellulose derivative, Biochemical activities

## Abstract

Nanoparticles (NPs) formulation in biopolymers is an attractive process for the researcher to decrease the disadvantages of NPs application alone. Bimetallic NPs are a promising formula of two NPs that usually act as synergetic phenomena. Zinc oxide and gold NPs (ZnO@AuNPs) biosynthesis as a bimetallic was prepared via the eco-friendly manner currently. Carboxymethylcellulose (CMC) was employed for the formulation of ZnO@AuNPs as a nanocomposite via a green method. Physicochemical and topographical characterization was assigned to ZnO@AuNPs and nanocomposite features. The nanostructure of bimetallic NPs and nanocomposite were affirmed with sizes around 15 and 25 nm, respectively. Indeed, the DLS measurements affirmed the more reasonable size and stability of the prepared samples as 27 and 93 nm for bimetallic NPs and nanocomposite, respectively. The inhibitory potential of nanocomposite was more than ZnO@AuNPs against *Staphylococcus aureus, Escherichia coli*, *Salmonella typhi*, *Enterococcus faecalis, Mucor albicans*, *Aspergillus flavus*, and *Mucor circinelloid*. ZnO@AuNPs and nanocomposite exhibited antioxidant activity via DPPH with IC_50_ of 71.38 and 32.4 µg/mL, correspondingly. Excellent anti-diabetic potential of nanocomposite with IC_50_ of 7.4 µg/mL, and ZnO@AuNPs with IC_50_ of 9.7 µg/mL was reported compared with the standard acarbose with the IC_50_ of 50.93 µg/mL for amylase inhibition (%). Photocatalytic degradation of RR195 and RB dyes was performed by ZnO@AuNPs and nanocomposite, where maximum degradation was 85.7 ± 1.53 and 88.7 ± 0.58%, respectively using ZnO@AuNPs, 90.3 ± 0.28 and 91.8 ± 0.27%, respectively using nanocomposite at 100 min.

**Graphical Abstract:**

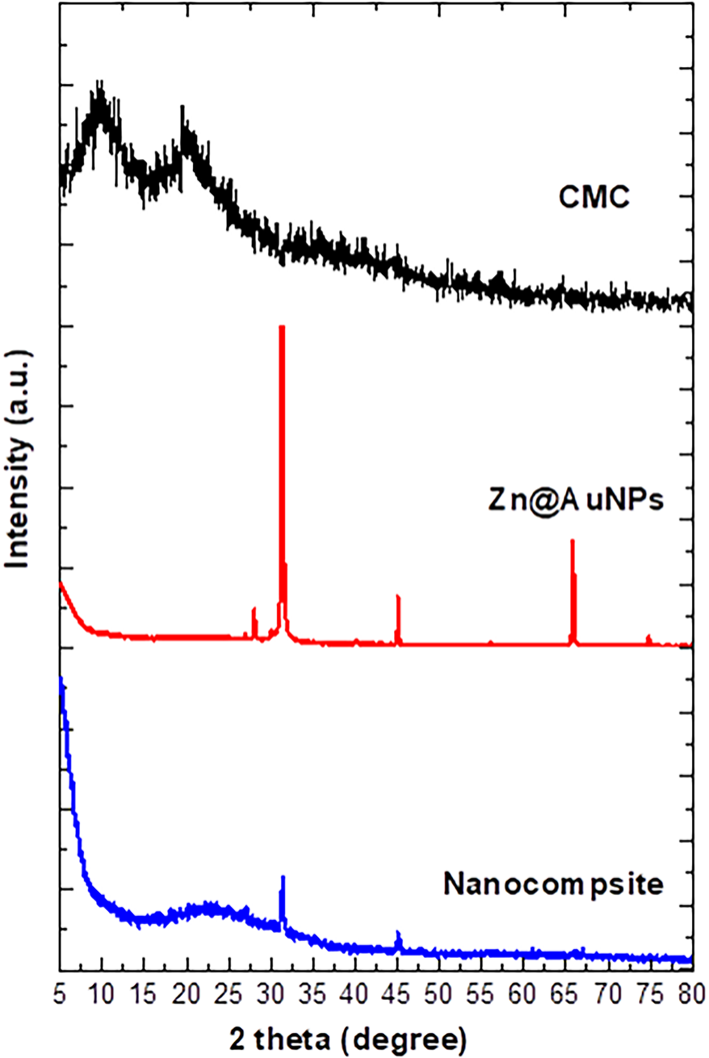

## Introduction

Biopolymers are a large group of natural polymers that are formulated inside living organisms (Yahya et al. 2022). Cellulose is the majority abundant biopolymer on the earth that is basically produced in plants and some kinds of bacteria (Hasanin 2022). In polar and nonpolar solutions, cellulose does not dissolve, and it is clear that water does not either. A vast set of cellulose derivatives, which are split into ether and ester cellulose derivatives, may be created by performing several changes on plain cellulose. Carboxymethylcellulose (CMC) is the most promising cellulose derivative, it is currently employed in a wide range of cutting-edge application domains, including biomedical engineering, wastewater treatment, food, paper, textile, and pharmaceutical industries, as well as energy generation and storage. This is because of the material's unique surface qualities, mechanical strength, adjustable hydrophilicity, viscous characteristics, availability and quantity of raw ingredients, low-cost synthesis technique, as well as a number of other factors. Numerous research articles have covered CMC, depending on their sources and application areas. Consequently, there is a great demand for a comprehensive and well-organized review that can provide a current and in-depth examination of CMC (Abdelhameed et al. 2023). Otherwise, metal nanoparticles (NPs) are widely used as promising drugs and biological agents as well as precursors for several industrial applications (Abdelghany et al. 2018; Rashid et al. 2021). Several polymers have been applied as support and stabilizing agents for various metal NPs such as Zinc oxide nanoparticles (ZnONPs) and gold NPs (AuNPs). Using different metal nanoparticles (NPs) in combination with biopolymers to create multifunctional nanocomposites is an intriguing way to broaden the range of applications for these materials. ZnONPs were recommended by form US FDA as a substance that is “Generally Recognized as Safe” (GRAS) and have a low level of toxicity for humans. The other used metal in the present investigation is gold (Au), Au has long been researched for its possible use in medicine. Recent investigations have shown that AuNPs offers various advantages over other NPs. This is mostly due to highly optimised manufacturing techniques that produce distinctively shaped and sized AuNPs with unique characteristics. The capacity to modify the surface of nanogold particles with different targeting and functional chemicals considerably broadens the range of their potential therapeutic uses, with an emphasis on cancer therapy (Ameen et al. 2023). Functionalized AuNPs are especially promising prospects for usage as the basis of innovative treatments because to their good biocompatibility and predictable bio-distribution patterns (Sztandera et al. 2019).

Numerous physical, chemical, and biological processes can be used to create NPs. A few examples of physical methods to create ZnONPs are vapor deposition, arc plasma, thermal evaporation, etc. Chemical methods include the sol–gel process, precipitation, and hydrothermal procedures ZnONPs (Siddiqi et al. 2018).

Biological techniques of NPs synthesis are relatively new and can make use of microbes and plant extracts (Alghonaim et al. 2024; Al-Rajhi et al. 2022a; Abdelghany et al. 2023a).The creation of metal NPs was performed via biological process using algae, plants, bacteria, fungi, and yeast. Some products were secreted by these biological sources used as reducing agents for NPs synthesis (Abdelghany et al. 2018; Saravanan et al. 2021). Fungi consider excellent source for NPs synthesis, due to rate of their growth compared to other microorganisms, cheap growth substrate, and capability to tolerate stress condition as well as their metabolites which stimulate the creation of NPs. Several investigations reported the ability of fungi to create gold, copper, titanium and silver, selenium NPs. According to performed investigations, the bacteriostatic or bactericidal properties of NPs resulted from physio-chemically interaction among the bacteria and NPs (Al-Rajhi et al. 2022b). Several factors such as composition, shape, size, crystallinity, surface modification, defects, and dose of NPs, and type of tested bacteria may determine the bactericidal activity (Abdelghany et al. 2023a; Gogurla et al. 2014). Al-Rajhi et al. (2022b) and Piktel et al. (2021) mentioned that commercial applications of NPs may enhance and extend via doped of NPs with polymers. ZnONPs has been proved more efficient against wide range of microorganisms compared to other NPs (Qanash et al. 2023). AuNPs are fewer investigated for their antimicrobial influence compared to other NPs, besides the little information about the action mechanisms of these NPs (Dediu et al. 2022). Jiménez et al. (2015) mentioned that ZnO and AuNPs were independently tested against pathogenic microorganisms but few studies documented the activity of bimetal NPs mixture.

NPs were applied recently as a photocatalyst for dyes degradation as well as water treatment. In the present investigation, the created bimetallic NPs and nanocomposite were applied for dyes degradation, from which Rhodamine B (RB) and Reactive Red 195 (RR195). RB is belongs to the group of xanthene dye, and commonly utilized in paper, food, printing, textiles, pharmaceuticals, and laser materials. Skin, respiratory disorders and eye irritation may arise as exposure to this dye. Because of this problem that generated by RB, it is essential to eliminate these dyes from environment (Ahmad et al. 2021). In general, reactive dyes are the most problematic since they frequently slip through standard treatment systems unharmed (Pérez-Calderón et al. 2020). RR195 is extensive use in the ink of textile and paper industries. The process of photocatalysis take place via the photochemical reaction at the metal oxide semiconductor surface, this reaction includes at minimum two steps taking place concurrently, the first step represents oxidation began by photo-stimulated positive holes, and the 2nd step represents reduction began by photo- stimulated negative electrons (Alakhras et al. 2020). The multi-functionalization application of bimetallic NPs attracted the attention of many researchers, therefore the developed bimetallic NPs via create a nanocomposite based on the bimetallic ZnO@AuNPs was the goal of this study with biological and environmental applications.

## Materials and methods

### Materials

Sigma Chemical Co., Ltd (St. Louis, MO, USA) was the source of the following chemicals: Carboxymethyl cellulose with properties: 5–10% of H_2_O; 6.5–7.5 of pH (1% in water); purity is > 99.5%, and mean substitution degree of 0.79. Zinc acetate and Gold (III) chloride trihydrate. Two dyes including Reactive Red 195 (RR195) and Rhodamine B (RB). All microbial media and reagents in analytical grade forms were obtained from Loba Chem., India.

## Methodology

### Mycocreator of nanoparticles

The bio-creator of NPs in our investigation was *Penicillium crustosum* that isolated from soil sample of industrial city of Jazan, Saudi Arabia. The isolate was identified morphologically and genetically with accession number OM836435.1in the Gene Bank. At 30 ℃ for 6 days, *P. crustosum* was cultivated on slant containing Czapek Dox Agar medium followed by preservation at 5 ℃ in the refrigerator until further utilization.

### Biosynthesis of bimetallic nanoparticles

Free cell filtrate of *P. crustosum* medium was used as a capping and stabilizing agent to prepare bimetallic NPs. Briefly, 5.0 mM of zinc acetate was mixed with 5.0 mM of gold chloride and completely mixed in 100 mL of the above-prepared filtrate. The previously prepared mixture was stirring at 1500 rpm for 1 h at 25 ℃ (Room temperature). The collected solution was ultrasonicated in an ultrasonic water bat at 70 oC for 1 h. To affirm the biosynthesis of ZnO@AuNPs the reddish-whit color was consistent. Finally, biosynthesized bimetallic ZnO@AuNPs were clarified by centrifugation at 10 k rpm for about 10 min. The biosynthesis bimetallic nanoparticle was dilated in dialysis bags for 24 h using deionized water that changed each 4 h. Afterward, the collected solution was lyophilized and preserved in a refrigerator for further use.

### Formulated of bimetallic nanoparticle

Bimetallic NPs formulation was carried out using 100 mL of 1% (w/v) CMC/0.1 g of bimetallic. In detail, the formulation of bimetallic was carried out using an ultrasonic probe. The above-prepared mixture was ultrasonicated in the ice bath for 10 min and the homogenous solution was collected and lyophilized for further uses.

### Characterizations

The comparative study of the biosynthesis nanoparticle used physiochemical analysis, including Ultra Vilote-Visble Spectroscopy (UV–Vis) (V-630 UV–Vis spectrophotometer, Jasco, Japan) in the range of 1000–200 nm, Fourier transfer infrared spectroscopy (FTIR) (Nicolet Impact-400 FT-IR spectrophotometer) in the range of 400–4000 cm^–1^, and x-ray diffraction (XRD) that was examined utilizing a Diano X-ray diffractometer (Philips). The crystal size calculations were made according to Debye Scherrer’s (Al-Mamun et al. 2021). Else, the topographical experiment comprised field emission scan electron microscopy (SEM) Model Quanta 250 FEG attached with EDX unite and transmission electron microscopy (TEM) Model JEM2010, Japan attached with selected area x-ray diffraction (SAED). The DLS measurements included average particle size distribution (PS), and polydispersity index (PDI) and average zeta potential were measured via Nano-ZS, Malvern Instruments Ltd., UK.

### Antimicrobial activity

ZnO@AuNPs and their nanocomposite were tested against different microorganisms including *Salmonella typhi* (ATCC 6539), *Enterococcus faecalis* (ATCC 10541)*, Escherichia coli* (ATCC 8739)*, Staphylococcus aureus* (ATCC 6538)*, Candida albicans* (ATCC 10221)*, Mucor circinelloid (AUMMC 11656)* and *Aspergillus flavus* (RCMB 02782) via well diffusion method according to Alawlaqi et al. (2023) with minor changes. The tested species of bacteria/fungi were inoculated by streaking technique on the nutrient/potato dextrose agar media. In each well, 100 μL of ZnO@AuNPs and nanocomposite, as well as standard amoxicillin as antibiotic and clotrimazole as antifungal, at dose of 1000 μg/mL were added. The inoculated plates were kept in refrigerator for 30 min before incubation. At the end of incubation period (incubated for 24 h/72 h at 37 °C/28 °C, for bacteria/fungi), the diameter of the appeared inhibition zone was recorded.

### Assessment of minimum inhibitory concentration (MIC) and minimum bactericidal concentration (MBC)

According to the CLSI, the microdilution technique was used to calculate the MIC. All of the wells on the microdilution plates were first filled with Müeller-Hinton broth. Later, concentrations of the ZnO@AuNPs and nanocomposite were added. To achieve a final concentration of 2 × 10^5^ CFU/mL, the bacterial suspensions were adjusted to 0.5 on the McFarland scale, diluted, and placed in the wells. The plates were then incubated for 24 h at 37 2 °C. Through a spectrophotometric analysis at 620 nm, MIC of the standard medication that may suppress microbial growth was identified. Following the MIC data, MBC was established. Each well with no discernible bacterial growth was given an aliquot of 10 µL, which was then aseptically removed and planted on Müeller-Hinton agar. The plates were incubated for 24 h at 35 °C. MBC had the lowest concentration after this incubation, when no microorganisms grew (French 2006).

### Antioxidant activity

The free radical scavenging potential of the dilute ZnO@AuNPs was assayed via a technique of 1,1-diphenyl-2-picryl hydrazyl (DPPH). The utilized DPPH was prepared by its dissolve in methanol (24 mg/100 mL) for stock solution creation. The prepared stock solution was filtrated, and then 3 mL of DPPH were added to 100 µL of different dilutions of tested compounds in test tubes followed by keeping under darkness condition for 30 min. The wave length of the reaction mixture was recorded at 517 nm. The following equation was applied to calculate the % of antioxidants.

$$\mathrm{Antioxidant \,activity \% }=\frac{{\text{CRA}}-{\text{TRA}}}{{\text{CRA}}}\times 100$$

where: CRA, Control reaction absorbance; TRA, Treatment reaction absorbance. DPPH solution in methanol was used as a standard. Ascorbic acid was used as positive control (Abdelghany et al. 2023b).

### Photo-catalytic degradation

Degradation of dyes (RR195 and RB) was performed to detect the photo-catalytic potential of ZnO@AuNPs and nanocomposite under different conditions. At different times ranging from 20 to 140 min with shaking processes, 100 μg/mL of ZnO@AuNPs and ZnO@AuNPs nanocomposite were separately mixed with 100 mL of each dye (10 mg/mL) to perform the dyes degradation in the presence of sunlight conditions. At the end of each time, the reaction mixture was centrifuged for 4.0 min at 10000 rpm. The obtained supernatant at (λmax) 538 nm and 554 nm were measured for RR195 and RB dyes degradation, respectively via UV–Vis spectroscopy (JENWAY 6305 Spectrophotomete). The efficacy of dye decolorization (%) was estimated according the following formula:

$${\text{Efficiency degradation \% }} = \frac{{{\text{C}}0{ }{-}{\text{ Cf}}}}{{{\text{C}}0}} \times { }100$$

where C0 is the initial absorbance, while Cf is the final absorbance.

At different temperatures ranged from 10 to 50 ℃, the photo-catalytic potential of ZnO@AuNPs and nanocomposite to dyes degradation at the optimum period from previous test was estimated. At different concentrations of dyes ranged from 5 to 25 mg/mL, the photo-catalytic potential of ZnO@AuNPs and nanocomposite to dyes degradation at the optimum period and temperature from previous test was estimated.

### Statistical analysis

Standard deviation was recorded for three replicates of outcomes as well as SPSS version 25, Minitab version 19, and version 365 of Microsoft excel at 0.05 level of probability were applied for statistical estimation. The analysis of variance, one-way ANOVA, and post hoc Tukey’s test were utilized to investigate quantitative result with a parametric distribution. The confidence interval was set to 95% and the margin of accepted error was fixed to 5%.

## Results and discussion

**Fig. 1:**
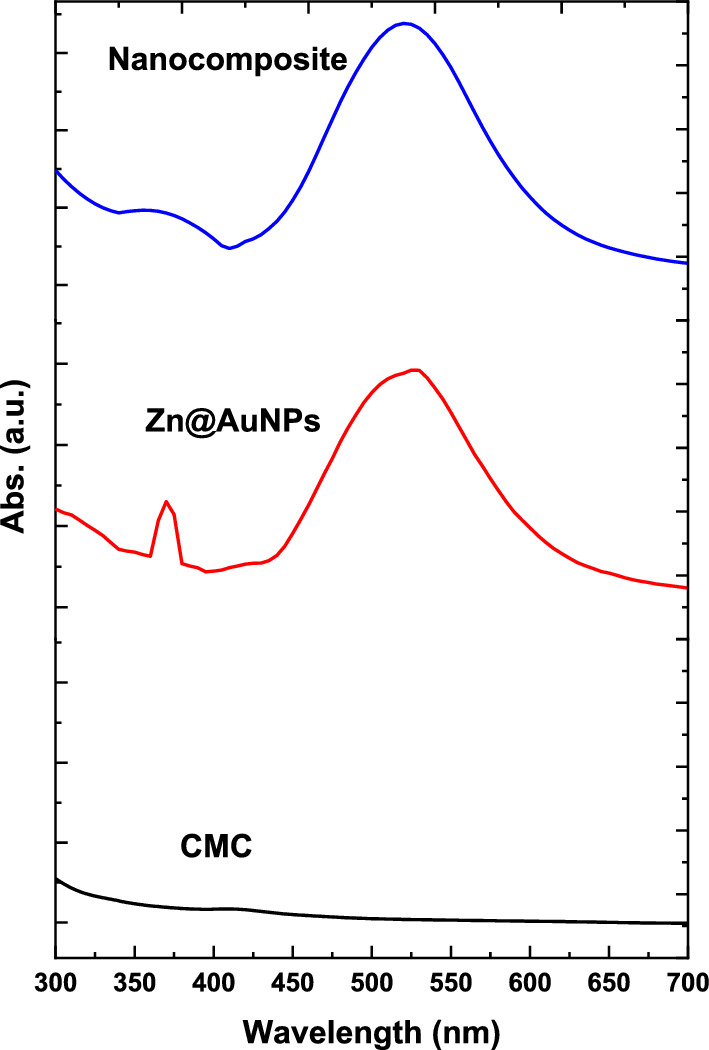
UV–vis of CMC, bimetallic nanoparticles and nanocomposite

Supplementary (1) showed GC/MS analysis of free cell filtrate of *P. crustosum* medium, where numerous constituents that may be plays an important role as reducing agents. These constituents including 2-(dimethylamino)-3-phenylbenzo[b]thiophene, isochiapin b, 11-octadecenal, 1-fluoro-3-(1-phenylethyl)benzene, methyl oleate o-isopropylidene, isopropyl myristate, methyl hexadecanoate, 2,3-dihydroxypropyl palmitate, 9-octadecenoic acid (z)-, glycidyl palmitate, 3',4',7-trimethylquercetin, ethyl iso-allocholate, and campesterol. Characterizations of biosynthesis bimetallic NPs were carried out via a physicochemical and topographical analysis. Physicochemical analysis included UV–vis, FTIR and XRD. Figure 1 shown the UV–vis spectra of CMC, Zn@AuNPs and nanocomposite. CMC spectrum show non-significant peaks (Yan and Chai 2021). Otherwise, the bimetallic shown two obvious peaks at 371 and 523 nm referred to ZnO and Au NPs, respectively with nonsignificant shifting in comparison with other literatures (Sun et al. 2011; Guo et al. 2014) that due to interact between two NPs during synthesis. Moreover, the nanocomposite spectrum was performed a broad two peaks at 368 and 518 nm were referred to bimetallic NPs. Indeed, the SPR was affected by the formulation of bimetallic that represented broadness in the peaks according to the surface interaction that could be effect the electronic cloud and by default effect SPR this conclusion is in a nice agreement with other literatures (Jana et al. 2016). However, the broadness of peaks was due to involved of nanoparticle into CMC chains that reduced the promotion of electrons to high energy in comparison with the free ones.

**Fig. 2:**
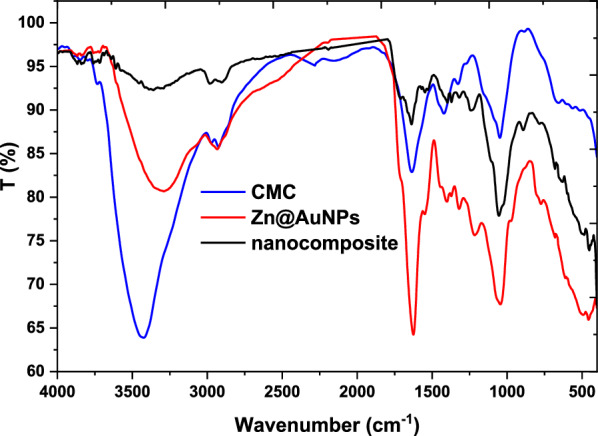
FTIR of CMC, bimetallic nanoparticles and nanocomposite

The FTIR spectra of CMC, ZnO@AuNPs, and nanocomposite are illustrated in Fig. 2. The CMC spectrum was performed, and a significant band was referred to as pure CMC at 3428, 2930, and 1780 cm^−1^. These bands were attributed for -OH stretching bands, CH stretching vibration, and a small band of the carboxylic acid's C = O, respectively.

In addition, bands at 1641 and 1425 cm^−1^ were corresponded to carbonyl (COO-) group's symmetric and asymmetric stretching vibration modes (Cuba-Chiem et al. 2008). Moreover, the carbohydrate band was assigned at 1046 cm^−1^ (Elashmawi and Al-Muntaser 2021). Otherwise, the bimetallic NPs were assigned bands at 3291, 2923, 1619, 1540, 1398, 1214, and1035 cm^−1^ were referred to as bending vibrations of amines, CH and CH_2_ aliphatic bending groups, C-N stretching vibration of aromatic rings, N–O starching, C-H bending, C-N starching, and C–O–C polysaccharide, respectively (Hasanin 2022). Additionally, bands at 610 and 500 cm^−1^ referred to AuNPs (Wang et al. 2012) and ZnO nanostructures (Hakim et al. 2020), respectively. On the other hand, the nanocomposite spectrum was shown a significant differences involved in reduce of intensity of OH band and CH band was spitted. As well as the Au and ZnO nanostructure was assigned with smallest intensity in comparison with the bimetallic spectrum. These observations were strongly affirmed the UV–vis study conclusion.

**Fig. 3:**
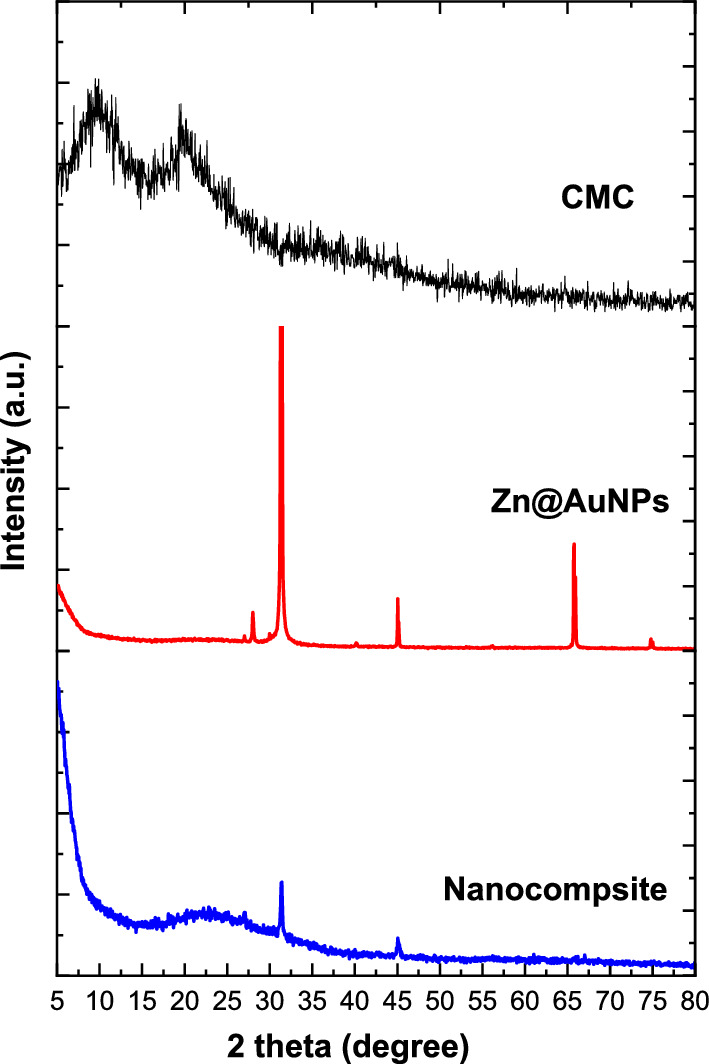
XRD patterns of CMC, bimetallic nanoparticles and nanocomposite

Additionally, Fig. 3 displays the crystallographic pattern of the prepared nanocomposite and its neat material. The pure CMC pattern was shown with two hub peaks at 9.8 and 20° which were referred to as the amorphous structure of the CMC (Abdelhameed et al. 2023). The bimetallic pattern was observed with peaks of ZnONPs at 28, 31.3, 45, and 56^o^ (Venkatesan et al. 2019) and AuNPs at 40, and 74 ^o^ (Peng et al. 2008). This means that the metal not fusion but take placed via attraction the particles to each other according to each metal was presented individual in the XRD pattern. On the other side, the nanocomposite presented a less crystalline structure in comparison with a bimetallic pattern with a peak at 22^o^ that referred to CMC as ahub due to the interaction of NPs with the molecular structure of CMC chains. Furthermore, the peaks at 27, 31, and 45 ^o^ were referred to as ZnONPs and small peaks at 40^o^ and 70 ^o^ were referred to as AuNPs with a significant difference in comparison with the neat bimetallic NPs pattern. Moreover, the crystals size were calculated according to Debye Scherrer’s and observed as 10 and 75 nm for bimetallic and formulated nanocomposite, respectively. Also, these observations were due to the trapped of NPs into CMC chains.

**Fig. 4:**
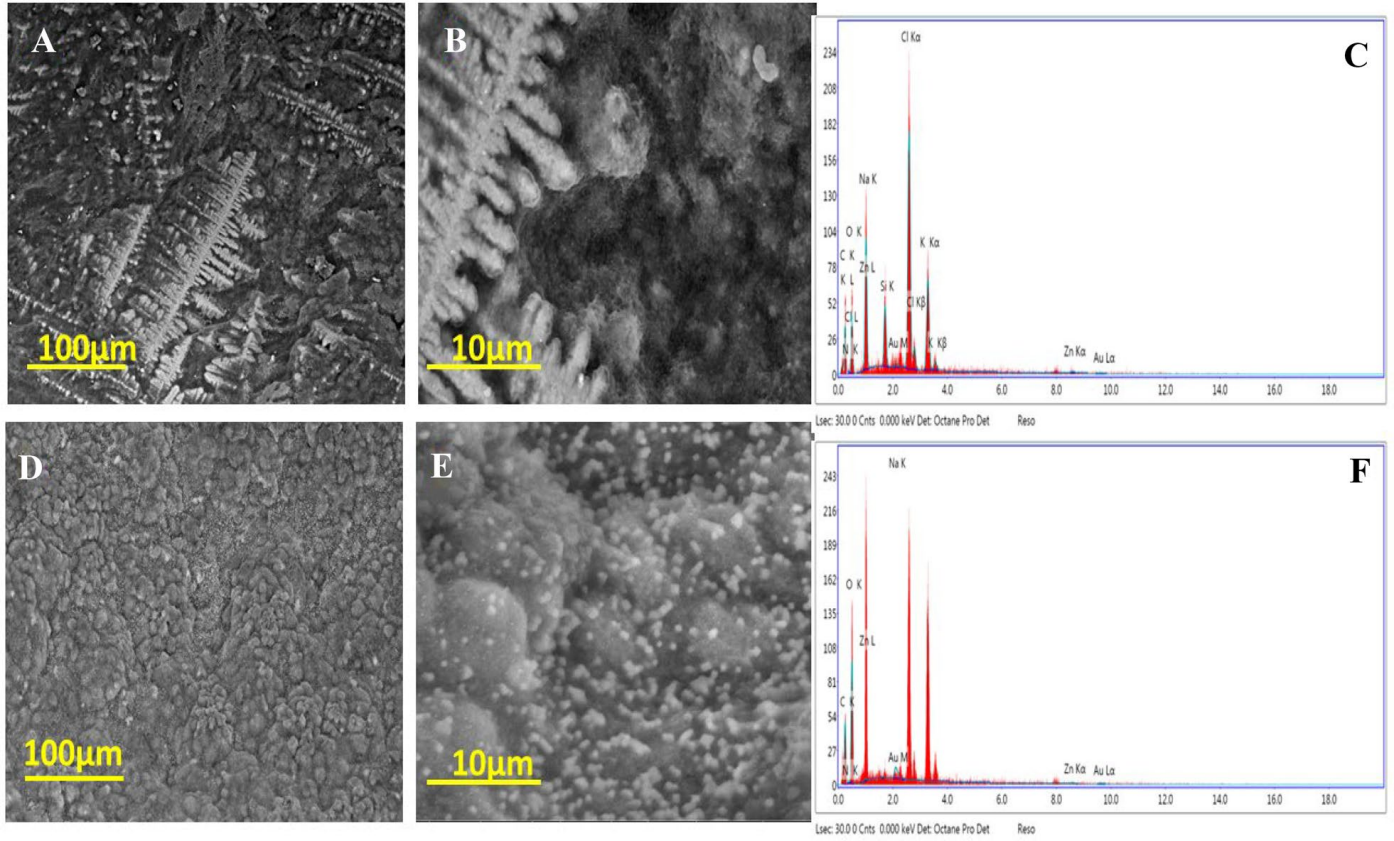
SEM images of bimetallic with low and high magnifications (**A** and **B**, respectively) as well as nanocomposite with low and high magnifications (**D** and **E**, respectively) and EDX charts of bimetallic nanoparticles (**C**) and nanocomposite (**F**)

The morphological study was carried out using two tools of topographical analysis including SEM and TEM. Figure (4) was illustrates the SEM images of bimetallic NPs and nanocomposite as well as the EDX charts of both as well. The low magnification SEM image of bimetallic NPs (Fig. 4A) was observed as a rods shape with some aggregations. Otherwise, the high magnification image (Fig. 4B) was clarified these rod were consisted of small spherical particles. Additionally the EDX chart (Fig. 4C) was affirmed the presence of carbon, oxygen, nitrogen, zinc and gold atoms with some impurities like chlorine, potassium, silicon, and sodium which due to the fungal medium extract protein and carbohydrates remaining. Our results were agreement with Nehru et al. (Nehru et al. 2023) using the fungi for synthesis of NPs. However, the nanocomposite low magnification image (Fig. 4D) was observed a unique surface structure related to CMC that lustrous with a spherical particle that referred to bimetallic particles, which appeared clearly via the high magnification image (Fig. 4E). The EDX chart of nanocomposite presented with carbon, oxygen, nitrogen, sodium, zinc, and gold that were relevant to CMC and bimetallic elemental composition.

**Fig. 5:**
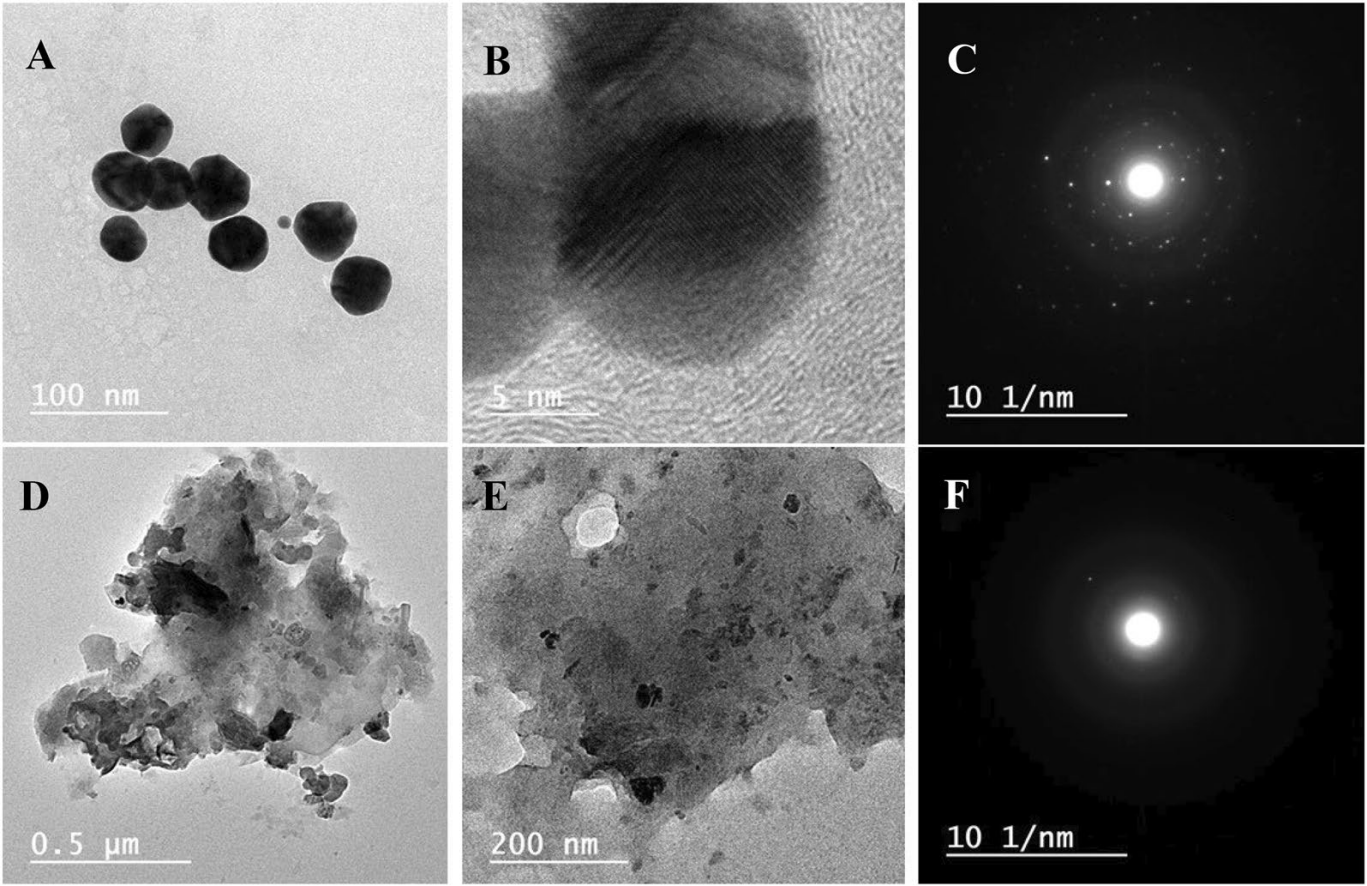
TEM images of bimetallic with low and high magnifications (**A** and **B**, respectively) as well as nanocomposite with low and high magnifications (**D** and **E**, respectively) and SAED pattern of bimetallic nanoparticles (**C**) and nanocomposite (**F**)

TEM images as well as SAED were presented in Fig. 5. The bimetallic images with low (Fig. 5A) and high (Fig. 5B) magnifications illustrated a clear spherical nanostructure with an average size of around 15 nm that in a nice agreement with the SEM study conclusion. Moreover, the SAED affirmed the high crystallinity behaviors of bimetallic NPs (Fig. 5C) as well as recorded previously (Rashid et al. 2021). On the other side, the nanocomposite was shown as an aggregated nanostructure containing small irregular spherical particles referred to as ZnO@AuNPs formulated in a CMC matrix in a TEM image at low magnification (Fig. 5D). The irregulatbility of the ZnO@AuNPs could be due to the interaction of some parts of CMC and bimetallicas well as reported in the study of El-Naggar et al. (2022). The TEM image at high magnification (Fig. 5E) emphasized the formulation of bimetallic NPs into CMC via a nanostructure with an average particle size of 25 nm. In addition, the SAED pattern illustrated a low crystallinity in comparison with the neat bimetallic due to the CMC crystallography nature also (Fig. 5F), these observations strongly agree with the XRD study conclusion.

DLS measurements including PS, PDI, and Zeta of ZnO@AuNPs and nanocomposite were presented in Table 1. The ZnO@AuNPs size was recorded as 27 nm with PDI 0.16 that referred to stable colloidal solution. Moreover, the average zeta potential was recorded as − 32 mV which referred also to high stability. In addition, the nanocomposite recorded particle size 93 nm with PDI 0.23 also related to high stabilized and affirmed with average zeta potential − 22 mV. Indeed, the size of particles of ZnO@AuNPs and nanocomposite were recorded in TEM as smaller which could be due to the aggregation of particles in the colloidal state.

**Table 1:** Shows zeta potential measurements prepeared samples

	Average particle size/nm	PDI	Av. Zeta potential, mV
ZnO@AuNPs	27	0.16	− 32
Nanocomposite	93	0.23	− 22

**Fig. 6:**
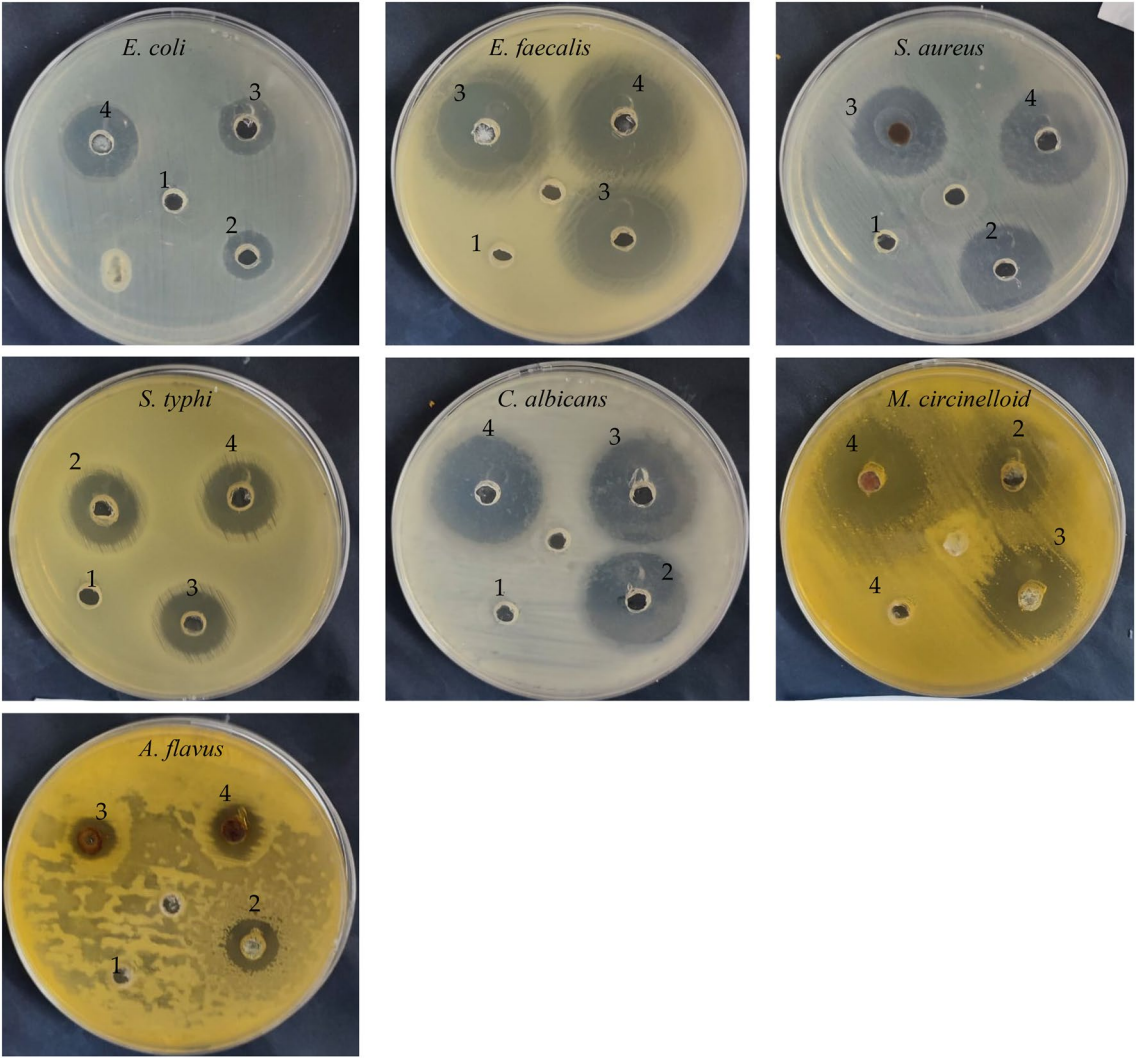
Inhibitory action of tested compounds against tested microorganisms (1, solvent used of tested compounds; 2, positive control Gentamycin/Nystatin; 3, ZnO@AuNPs; 4, nanocomposite)

The antimicrobial activity of ZnO@AuNPs and nanocomposite against different microorganisms including bacteria, unicellular yeast, and filamentous fungi was recorded (Table 2 and Fig. 6). ZnO@AuNPs and nanocomposite exhibited antimicrobial activities but with different inhibition zones, however, more inhibition zones (23, 19, 20, 26, 28, 15, and 29 mm) were observed using nanocomposite compared to the inhibition zones (22, 15, 17, 25, 25, 14, and 23 mm) using ZnO@AuNPs against *S.aureus, E. coli*, *S. typhi*, *E. faecalis, C. albicans*, *A. flavus*, and *M.circinelloid*, respectively. These results were compared to standard antibiotic/antifungal agents, surprisingly nanocomposite reflected high antimicrobial activity against all tested microorganisms, as well as ZnO@AuNPs against three tested microorganisms compared to antibiotic/antifungal drugs. Within this context**,** MIC of the nanocomposite was less than the MIC of ZnO@AuNPs against *E. coli*, *S. typhi*, and *E. faecalis*, but it was similar in case of the *S.aureus* and *C. albicans*. Also, MBC of nanocomposite was less than the MBC of ZnO@AuNPs against *S.aureus, E. coli*, *S. typhi*, and *E. faecalis*. MBC/MIC index nanocomposite was less than 4 indicating its bactericidal properties against *S.aureus, E. coli*, *S. typhi*, *E. faecalis*, and *C. albicans*. Except for *E. coli*, ZnO@AuNPs exhibited bacteriostatic potential against tested microorganisms via calculation MBC/MIC index (Table 2). Raghupathi et al. (Raghupathi et al. 2011) attributed the antimicrobial activity of ZnO NPs to the generation of reactive oxygen species besides the deposit in the cell cytoplasm. From the result of Doghish et al. (Doghish et al. 2022), CMC-AuNPs demonstrated antimicrobial activities but with different levels of activities depending on the tested microorganism, for instance, *Bacillus cereus* and *S. aureus* were inhibited with MIC value of 25 μg/mL, *Klebsiella oxytoca* with MIC value 50 μg/mL, and *Escherichia coli* with MIC value of 100 μg/mL. Moreover, *A. fumigatus*, *A. niger*, *A. terreus*, and *C. albicans* were inhibited by CMC-AuNPs at 500 μg/mL with inhibition zones 26, 23, 13, and 20 mm, respectively. Our findings document that nanocomposite has greater effectiveness than ZnO@AuNPs, and therefore the present results encourage the application of ZnO@AuNPs and particularly nanocomposite in the biological activity. According to several studies, the improvement of hybrid composites has markedly developed since they presented a broad range of applications, which include antimicrobial activities, treatment of water, and photocatalysis (Shi et al. 2020).

**Table 2:** Inhibitory action of ZnO@AuNPs and nanocomposite against tested bacteria and fungi with MIC, MBC, and MBC/MIC index detection

Tested microorganisms	Inhibition Zone (mm)				MIC (µg/mL)		MBC (µg/mL)		MBC/MIC Index	
	ZnO@AuNPs	Nanocomposite	+ ve C*	-ve C^a^	ZnO@AuNPs	Nanocomposite	ZnO@AuNPs	Nanocomposite	ZnO@AuNPs	Nanocomposite
*S.aureus*	22 ± 0.1	23 ± 0.3	20 ± 0.3	0.0	62.5	62.5	125	62.5	2.0	1.0
*E. coli*	15 ± 0.1	19 ± 0.2	14 ± 0.1	0.0	62.5	31.25	250	62.5	4.0	2.0
*S. typhi*	17 ± 0.2	20 ± 0.1	18 ± 0.2	0.0	125	31.25	250	31.25	2.0	1.0
*E. faecalis*	25 ± 0.4	26 ± 0.2	25 ± 0.1	0.0	31.25	15.62	62.5	15.62	2.0	1.0
*C. albicans*	25 ± 0.2	28 ± 0.1	24 ± 0.1	0.0	15.62	15.62	31.25	31.25	2.0	2.0
*A. flavus*	14 ± 0.2	15 ± 0.2	14 ± 0.2	0.0	–	–	–	–	–	–
*M.circinelloid*	23 ± 0.3	29 ± 0.4	20 ± 0.3	–	–	–	–	–	–	–

^a^ + ve C, positive control (Gentamycin/Nystatin) and -ve C, negative control (solvent of extraction used)

**Fig. 7:**
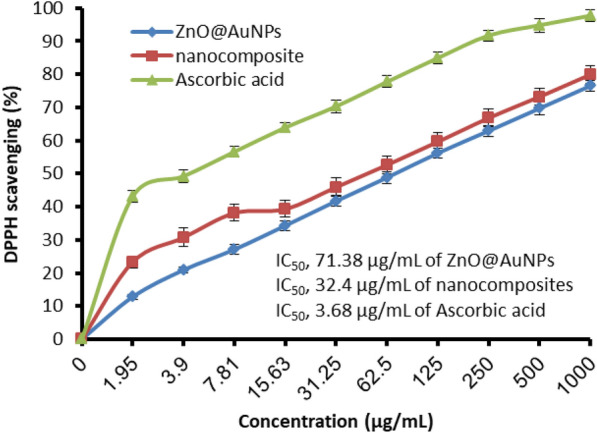
Antioxidant activity of ZnO@AuNPs and nanocomposite

There is a difference in the antioxidant activity between ZnO@AuNPs and nanocomposite at all tested concentrations (1.95–1000 µg/mL), where nanocomposite exhibited more antioxidant potential than ZnO@AuNPs (Fig. 7). At low concentrations (1.95, 3.90, 7.81, and 15.63 µg/mL), a remarkableDPPH scavenging % was recorded (23.3, 30.7, 38.1, and 39.3%, respectively) using nanocomposite compared to DPPH scavenging % (12.8, 20.9, 27.1, and 34.3%, respectively) using ZnO@AuNPs. This finding was reflected by less IC_50_ value (32.4 µg/mL) compared to the IC_50_ value (71.38 µg/mL) of ZnO@AuNPs. In context, the antioxidant activity of both ZnO@AuNPs and nanocomposite were compared to the authentic drug (ascorbic acid) that showed the IC_50_ value (3.68 µg/mL). The antioxidant activity of AuNPs and ZnONPs were applied alone and as bimetallic NPs which reflected different values of DPPH inhibition including 129.9, 105.0, and 76.07 µg/mL, respectively, moreover the bimetallic NPs exhibited good other biological activities such as anti-inflammatory and wound healing compared to the application of AuNPs or ZnONPs alone (Abd El-Aziz et al. 2023).

**Fig. 8:**
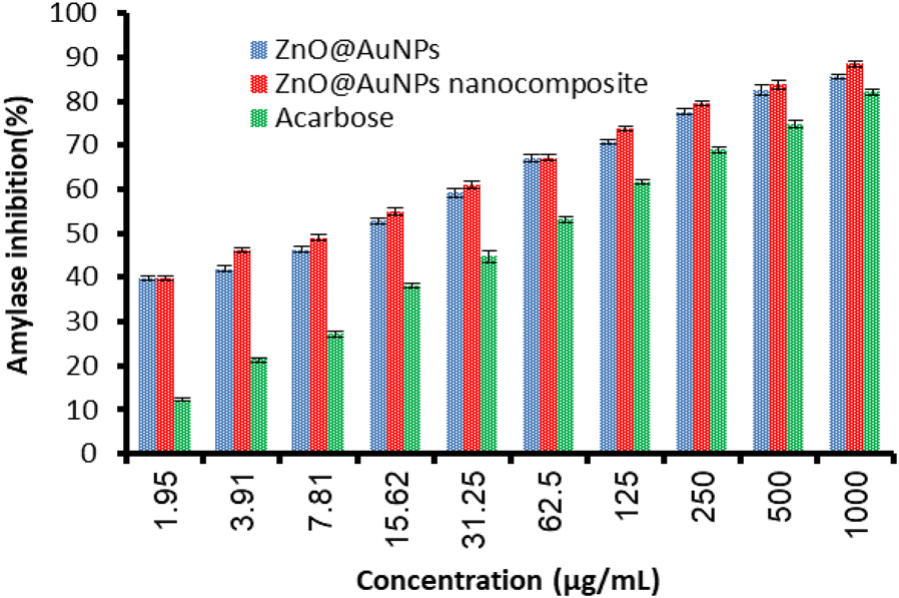
Amylase inhibition by ZnO@AuNPs, nanocomposite and acarbose

The anti-diabetic potential of ZnO@AuNPs and nanocomposite were recorded via evaluation of amylase inhibition (%) (Fig. 8). The inhibition of amylase % increased with increasing the concentration of tested ZnO@AuNPs and nanocomposite in dependent concentration style but nanocomposite exhibited more inhibition of amylase % at most tested concentrations. For instance, at 3.91, 15.62, 125, and 1000 µg/mL, the amylase % was 46.0, 55.0, 73.7, and 88.4%, respectively using nanocomposite, while it was 41.9, 52.7, 70.7, and 85.6%, respectively using ZnO@AuNPs. Surprisingly, ZnO@AuNPs and nanocomposite reflected more inhibition of amylase % compared to standard compound (Acarbose) at all tested concentrations with high IC_50_ value (50.9 µg/mL) compared with low IC_50_ value (7.4 µg/mL) of nanocomposite and IC_50_ value (9.7 µg/mL) of ZnO@AuNPs. The elevation of glucose concentrations in blood and hyperglycemia are affected mostly by the quantities of some produced oxidative enzymes including amyloglucosidase, α-amylase, and α-glucosidase. These enzymes promote the metabolic reactions responsible for the breakdown of polysaccharides into glucose and its metabolized rate (Robkhob et al. 2020). Velsankar et al. (2022) mentioned that the ZnONPs have been described as safe anti-diabetic and antioxidant agents that display a remarkable defensive role from the free radicals generation. Also, Ramachandran et al. (2019) and Badeggi et al. (2020) reported the application of AuNPs as an anti-diabetic agent. The application of ZnO NPs coated with Ag caused elevation of alpha-glucosidase and alpha-amylase enzymes inhibition with IC_50_ values of 24, and 47 µg/mL, respectively (Kumar et al. 2022). As mentioned in several studies, polysaccharides such as carboxymethyl cellulose stabilize NPs as well as raise their activity. The outcomes of our study indicated that the nanocomposite reflected good results compared to ZnO@AuNPs alone. Previously, Alric et al. (2013) reported the cytotoxic nil of ZnONPs and AuNPs due to rapidly eliminated via the kidneys. Therefore, the current investigation promotes the biological application of the created bimetallic NPs and nanocomposite.

The ability of ZnO@AuNPs and nanocomposite to removal two dyes RR195 and RB was recorded at different conditions. At different times ranging from 20 to 140 min, the decolorization percentages of both the two dyes were increment with time increasing up to 100 min with maximum degradation 85.7 ± 1.53 and 88.7 ± 0.58% for RR195 and RB, respectively using ZnO@AuNPs (Table 3). At the same time (100 min), nanocomposite caused maximum degradation of 90.3 ± 0.28 and 91.8 ± 0.27% for RR195 and RB, respectively. RB dye was degraded (78.6%) dye under solar light using ZnO doped Au NPs, this photocatalytic activity according to Yu and Kim (2021) perhaps due to the effect of the increment surface of Au NPs plasmon resonance. Au-metal oxide nanocomposites are characterized by a high surface-area-to-volume ratio besides their high stability when used as catalysts (Kauffman et al. 2019). Ahmad et al. (2021) documented the potential of Au doped ZnO for the degradation of the RB dye. The long time was accompanied by the release of dyes in the reaction solution; therefore a decline in its decolorization was occurred. RR 195 was degraded with a maximum rate of 91–94% after 70 min via ZnONPs as mentioned by Ali et al. (2021). Table 4 shows the effect of different temperatures on the photocatalytic degradation of dyes by ZnO@AuNPs and nanocomposite. High temperatures were suited for degradation compared to low temperatures, where at 40 and 50 ℃, the dyes degradation was more than at 10 and 20 ℃. The optimum temperature was 40 ℃, where the degradation was 86.2 ± 0.47% and 89.2 ± 0.25% using ZnO@AuNPs; 90.3 ± 0.58% and 91.7 ± 0.58% using nanocomposite for RR195 and RB, respectively. At 50 ℃, the rate of degradation decreased due to the weak adsorption forces, and declined in the connection between the adsorbent and the adsorbate. Moreover as mentioned by Malekkiani et al. (2022) the dissociation and solubility of dyes increase once the temperature rises and therefore the interaction between adsorbate and the adsorbent was minimized. The rate of degradation by ZnO@AuNPs and nanocomposite was decreased with increasing dyes concentration as illustrated in Table 5. At 5 mg/mL, the rate degradation % was 88.2 ± 0.29% and 91.8 ± 0.29% via ZnO@AuNPs, moreover, it was 94.2 ± 0.25% and 95.4 ± 0.55% via nanocomposite for RR195 and RB, respectively. While at 25 mg/mL, the rate degradation % was 77.2 ± 0.20% and 79.2 ± 0.20% via ZnO@AuNPs, moreover it was 81.2 ± 0.24% and 85 ± 0.50% via nanocomposite for RR195 and RB, respectively. It's clear that nanocomposite exhibited more dyes degradation than ZnO@AuNPs at all studied conditions.

**Table 3:** Photocatalytic activity of ZnO@AuNPs and nancomposite for dyes degradation at different times

Time	ZnO@AuNPs		Nanocomposite	
	RR195	RB	RR195	RB
20	19.17 ± 0.29e	21.67 ± 0.58e	25.16 ± 0.28e	29.33 ± 0.29 g
40	35.67 ± 0.58d	41.00 ± 2.00d	46.17 ± 0.29d	48.66 ± 0.58f
60	78.67 ± 1.53c	65.67 ± 1.04c	81.83 ± 0.29c	67.17 ± 0.28e
80	82.67 ± 0.57b	86.83 ± 0.29a	88.17 ± 0.76b	90.16 ± 0.29b
100	85.67 ± 1.53a	88.66 ± 0.58a	90.33 ± 0.28a	91.83 ± 0.27a
120	85.17 ± 0.29ab	82.00 ± 1.00b	87.00 ± 0.51b	89.17 ± 0.30c
140	78.00 ± 1.01c	80.16 ± 0.29b	82.33 ± 0.58c	85.16 ± 0.26d
HSD	1.19	1.23	0.57	0.43

**Table 4:** Photocatalytic activity of ZnO@AuNPs and nanocomposite for dyes degradation at different temperatures (At 100 min as optimum time)

Temperature℃	ZnO@AuNPs		Nanocomposite	
	RR195	RB	RR195	RB
10	37.17 ± 1.26d	41.83 ± 0.27d	46.17 ± 0.29d	48.33 ± 0.57d
20	40.16 ± 0.29c	44.17 ± 0.29c	55.40 ± 0.17c	66.23 ± 0.25c
30	83.53 ± 0.50b	83.16 ± 0.28b	87.23 ± 0.25b	89.34 ± 0.29b
40	86.16 ± 0.47a	89.23 ± 0.25a	90.33 ± 0.58a	91.67 ± 0.58a
50	85.84 ± 0.76a	88.83 ± 0.27a	89.50 ± 0.50a	92.17 ± 0.30a
HSD	0.95	0.36	0.51	0.54

**Table 5:** Photocatalytic activity of ZnO@AuNPs and nanocomposite for dyes degradation at different dyes concentration (At 100 min and 40 ℃ as optimum conditions)

Dye concentration mg	ZnO@AuNPs		Nanocomposite	
	RR195	RB	RR195	RB
5	88.17 ± 0.29a	91.83 ± 0.29a	94.23 ± 0.25a	95.37 ± 0.55a
10	86.23 ± 0.25b	89.23 ± 0.25b	90.21 ± 0.27b	91.83 ± 0.76b
15	85.23 ± 0.26c	87.50 ± 0.62c	88.20 ± 0.26c	89.90 ± 0.36c
20	82.82 ± 0.28d	85.13 ± 0.12d	86.40 ± 0.53d	88.23 ± 0.25d
25	77.20 ± 0.20e	79.20 ± 0.20e	81.23 ± 0.24e	85.00 ± 0.50e
HSD	0.33	0.44	0.43	0.66

## Conclusions

Green and eco-friendly methods were used for the biosynthesis of ZnO@AuNPs and formulated into nanostructure CMC. The produced nanocomposite was characterized via physicochemical and topographical techniques that affirmed the formulation of bimetalic NPs into CMC in the nanoscale. The created nanocomposite exhibited antimicrobial, antioxidant and anti-diabetic as well as photocatalytic degradation of dyes more than ZnO@AuNPs.

## Data Availability

Not applicable.
